# Supplementary material for: Content-rich biological network constructed by mining PubMed abstracts
Source: BMC Bioinformatics. 2004 Oct 8;5:147. doi: 10.1186/1471-2105-5-147 (PMC528731; doi:10.1186/1471-2105-5-147)
Supplement: Additional File 2 — The original results of the above study (non-essential files are deleted to keep the file size under the limit set by BMC bioinformatics). [file 1471-2105-5-147-S2.bz2 › chilibotAdditionalFile2/dip10/11ID8738158E56/html/PRNP_BCL2.html]

 


 **PRNP** and **BCL2** 
  
Found 12 abstracts in PubMed, retrieved 10.  
 

 What does Google say? 
 PDF only 
| .edu only 

---

**Interactive relationship** (e.g. stimulation, inhibition, etc)

**Stimulatory relationship**- Flupirtine normalizes the level of intracellular glutathione and increases the expression of the antiapoptotic Bcl 2  [ **BCL2** ]  protein in neuronal cells exposed to prion protein  [ **PRNP** ] .  Ref: 12532184 Drugs Today Barc , 2002
- Increase in Bcl 2  [ **BCL2** ]  and N myc occurred in Purkinje cells in CJD  [ **PRNP** ]  and OPCA.  Ref: 11585244 Acta Neuropathol Berl , 2001
**Neutral relationship**- In addition, we analyzed heteromeric interactions of Bcl 2  [ **BCL2** ]  with PrP  [ **PRNP** ]  and two Bcl 2  [ **BCL2** ]  related proteins, Bax and A1.  Ref: 8738158 Brain Res Mol Brain Res, 1996
- PrP  [ **PRNP** ]  may disrupt chains of Bcl 2  [ **BCL2** ]  molecules at the homomeric association site in the transmembrane region.  Ref: 8738158 Brain Res Mol Brain Res, 1996
- PrP  [ **PRNP** ]  associated with the carboxy terminus of Bcl 2  [ **BCL2** ]  amino acids 200 236.  Ref: 8738158 Brain Res Mol Brain Res, 1996
- Analysis of interaction sites in homo and heteromeric complexes containing Bcl 2  [ **BCL2** ]  family members and the cellular prion protein  [ **PRNP** ] .  Ref: 8738158 Brain Res Mol Brain Res, 1996
- The cellular prion protein  [ **PRNP** ]  PrP  [ **PRNP** ]  binds to the C terminus of Bcl 2  [ **BCL2** ]  but not Bax.  Ref: 8738158 Brain Res Mol Brain Res, 1996
- Regulation of Bcl 2  [ **BCL2** ]  and Bax expression also correlates with the survival effect elicited by PrP  [ **PRNP** ] .  Ref: 12676532 Mol Cell Neurosci, 2003
- Effect of flupirtine on Bcl 2  [ **BCL2** ]  and glutathione level in neuronal cells treated in vitro with the prion protein  [ **PRNP** ]  fragment PrP106 126.  Ref: 9344576 Exp Neurol, 1997

**Non-interactive relationship** (e.g. studied together, co-existance, homology, etc.)

- ... increased Bcl 2  [ **BCL2** ]  and N myc does not preclude per se cell death or death survival in CJD  [ **PRNP** ]  and OPCA.  Ref: 11585244 Acta Neuropathol Berl , 2001
- Expression of Fas, Fas ligand Fas L, ERK, MEK, Bcl 2  [ **BCL2** ] , Bax, N myc, c myc, pro caspase 2 and active caspase 3 was examined by immunohistochemistry in the cerebellum of six patients with sporadic CJD  [ **PRNP** ] , three patients with olivopontocerebellar atrophy OPCA and six age matched controls.  Ref: 11585244 Acta Neuropathol Berl , 2001
